# Gut Microbiome and Atherosclerosis: A Mendelian Randomization Study

**DOI:** 10.31083/j.rcm2502041

**Published:** 2024-01-29

**Authors:** Yue Li, Yunxian Chen, Zhe Li, Yanrong Li, Yicai Chen, Liangqiu Tang

**Affiliations:** ^1^Department of Cardiology, Yue Bei People's Hospital, Shantou University Medical College, 512099 Shaoguan, Guangdong, China; ^2^Department of Rheumatology and Immunology, Shenzhen People’s Hospital, The Second Clinical Medical College, Jinan University, 518071 Shenzhen, Guangdong, China

**Keywords:** coronary atherosclerosis, cerebral atherosclerosis, intestinal microbiota, phylum Firmicutes

## Abstract

**Background:**

According to recent studies, atherosclerosis and gut microbiota are related. Nevertheless, it has been discovered that the gut microbiota varies across studies, with its function still being debated, and such relationships not proven to be causal. Thus, our study aimed to identify the key gut microbiota taxa (GM taxa) at different taxonomic levels, namely, the phylum, class, order, family, and genus, to investigate any potential causal links to atherosclerosis.

**Methods:**

We employed summary data from the MiBioGen consortium on the gut microbiota to conduct a sophisticated two-sample Mendelian randomization (MR) analysis. Pertinent information regarding atherosclerosis statistics was acquired from the FinnGen Consortium R8 publication. To assess causality, the utilized principal analytical technique was the inverse variance-weighted (IVW) method. Supplementary to IVW, additional MR methodologies were employed, including weighted median, MR-Egger, weighted methods, and simple mode. Sensitivity analyses involved the application of Cochrane’s Q-test, MR-Egger intercept test, MR-PRESSO global test, and leave-one-out analysis.

**Results:**

Finally, after performing an MR study on the risk of 211 GM taxa on atherosclerosis, we discovered 20 nominal links and one strong causal link. Firmicutes (phylum ID: 1672) (odds ratio (OR) = 0.852 (0.763, 0.950), *p* = 0.004) continued to be connected with a lower incidence of coronary atherosclerosis, even after Bonferroni correction.

**Conclusions:**

Based on the discovered data, it was established that the phylum Firmicutes exhibits a causal relationship with a reduced occurrence of coronary atherosclerosis. This investigation could potentially provide novel insights into therapeutic objectives for atherosclerosis by focusing on the gut microbiota.

## 1. Introduction 

Amongst the array of afflictions categorized as cardiovascular diseases (CVD), atherosclerosis reigns as a prevailing force. Characterized by the accumulation of lipids and pronounced inflammation within pivotal arteries, atherosclerosis advances steadily, potentially resulting in dire clinical consequences, such as myocardial infarction (MI) and stroke. Despite its gradual nature and declining prevalence in certain nations, this insidious malady continues to claim a prominent position among the leading causes of mortality within our global community [1]. Atherosclerosis has emerged as a matter of international apprehension, and despite remarkable progress in comprehending the origin and management of ailments, there still exists an inadequacy in its prevention [2, 3]. The discovery of shielding or instigating elements in atherosclerosis carries paramount importance and necessitates further exploration into newly found treatment objectives.

The “microbiota” (referring to the myriad of bacteria, viruses, and fungi comprising the human gut) plays a vital role in maintaining a harmonious and flourishing human ecological system that abundantly thrives within the intestinal tract. Specifically, bacteria contribute to the processes of food digestion, fortify the immune system, and generate unique metabolites capable of permeating the host’s bloodstream [1]. Consequently, a more all-encompassing perception of metabolism is presently being proffered, asserting that modifications in the host’s metabolic processes, in conjunction with interactions between the gut microbiota and the host’s remote organs, collectively impact the entirety of human metabolism [4]. Mounting evidence suggests that the gut microbiota (GM) exerts regulatory control over the host’s immune responses, inflammatory processes, metabolism, and cardiovascular function [5]. For instance, it has been reported in some studies that the metabolites of gut microbiota, such as trimethylamine N-oxide (TMAO), short-chain fatty acid butyrate, and indole-3-propionic acid (IPA), have indirect influences on the occurrence of atherosclerosis [4, 6, 7]. Despite extensive epidemiological research, the underlying cause of the association between the composition of the gut microbiota and various diseases, such as CVD, remains largely elusive [8]. The intricacy arises from the presence of various supplemental factors, such as gender, age, and ethnicity, which can potentially impact both the progression of atherosclerosis and the composition of the gut microbiota. It poses a considerable challenge to adequately encompass these variables within an observational study, thereby limiting the capacity to definitively establish a causal relationship between the gut microbiome and atherosclerosis.

However, additional investigations are necessary to ascertain the precise role that various gut microbiota taxa (GM taxa) play in the development of atherosclerosis. Mendelian randomization (MR), a novel method for exploring the relationship between gut microbiota and atherosclerosis in this context, bears a resemblance to randomized controlled trials (RCT) [9]. Single nucleotide polymorphisms (SNPs) are referred to as instrumental variables (IVs) in MR investigations, serving to quantify the causal connection between exposure and the outcome of interest [10]. SNPs adhere to the concept that genetic variation is randomly assigned during meiosis, thereby circumventing confounding influences and potential reverse causation, thereby ensuring that relationships between genetic variants and outcomes remain unaffected [11]. Consequently, MR analysis can swiftly identify the causal link between a specific exposure and an outcome, more so than RCTs. Thus, to determine the potential impact of GM taxa on atherosclerosis in three distinct vascular locations: atherosclerosis (excluding cerebral, coronary, and peripheral arterial disease), coronary atherosclerosis, and cerebral atherosclerosis, we conducted MR analysis by utilizing the genome-wide association study (GWAS) summary statistics from the FinnGen and MiBioGen consortia. This approach may provide validation for existing evidence and yield novel insights pertaining to the treatment and prevention of atherosclerosis.

## 2. Materials and Methods

### 2.1 Study Design

**Fig. 1.**
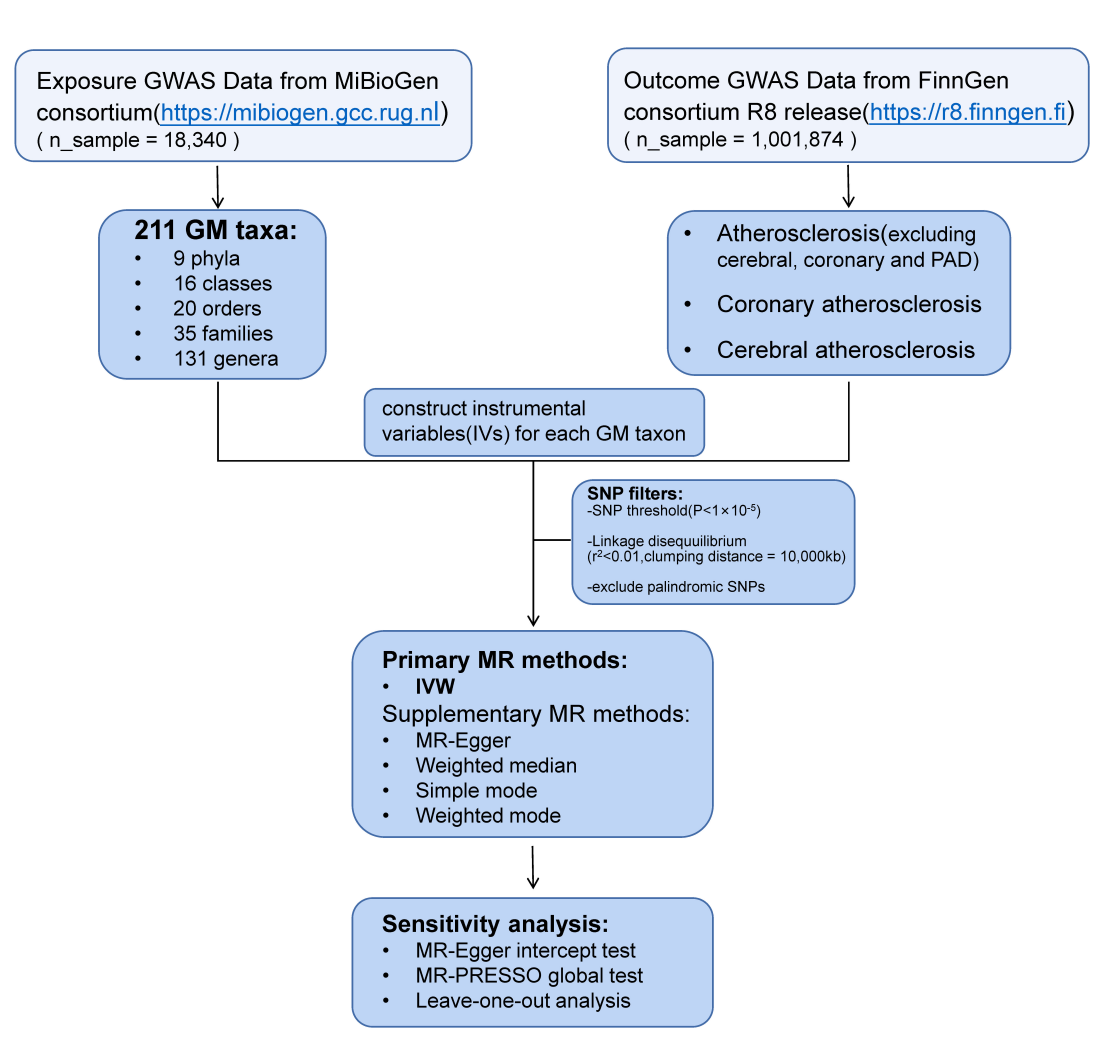
**Flowchart of the entire study**. Flowchart shows the general steps involved in our work, from the acquisition of data to the analysis methods used, which will be developed in detail below. GWAS, genome-wide association study; GM, gut microbiota; PAD, peripheral arterial disease; SNP, single nucleotide polymorphism; MR, Mendelian randomization; IVW, inverse variance-weighted.

Fig. 1 presents a comprehensive depiction of our research. Through the implementation of a two-sample MR analysis, we unveiled GM taxa that exhibit influential effects on atherosclerosis, specifically excluding cerebral, coronary, and peripheral arterial disease (PAD). Notably, our findings encompass the domains of coronary atherosclerosis and cerebral atherosclerosis. In reporting these findings, we have adhered to the guidelines of the esteemed strengthening the reporting of observational studies in epidemiology-MR (STROBE-MR) [12].

### 2.2 Data Source for Exposure

We obtained gut microbiota GWAS data from the genomic research on the gut microbiome by the MiBioGen consortium [13, 14]. This genome-wide association study delved into 211 transgenic taxa and encompassed 18,340 participants. Ultimately, it unveiled genetic variability associated with 131 genera, 35 families, 16 classes, and 9 phyla.

### 2.3 Data Source for Outcome

GWAS summary statistics for atherosclerosis, coronary atherosclerosis, and cerebral atherosclerosis were from FinnGen Release 8, one of the earliest personalized medicine initiatives to collect and analyze medical and genomic information from Finnish Biobank users, to identify links between genotype and phenotype (see https://www.finngen.fi/en) [15]. FinnGen uses the International Classification of Diseases (ICD) 10th Revision I70, I25.1, and I67.2 criteria to diagnose atherosclerosis, coronary atherosclerosis, and cerebral atherosclerosis, respectively. According to the I70 diagnostic criteria, atherosclerosis in our study included: atherosclerosis in the aorta, atherosclerosis in extremity arteries, atherosclerosis in the renal artery, generalized and unspecified atherosclerosis, and atherosclerosis in other arteries. Meanwhile, the following should be excluded: cerebral, coronary, mesenteric, pulmonary, and PAD. Table 1 summarizes the exposure and outcome in more detail.

**Table 1.** **Specific information regarding the outcome and exposure**.

	Cohort	Data source	Sample (N)	Case (N)	Control (N)
Exposure	211 GM taxa	MiBioGen	18,340	NA	NA
Outcome	Atherosclerosis	FinnGen (R8)	331,333	13,434	317,899
Outcome	Coronary atherosclerosis	FinnGen (R8)	328,042	42,421	285,621
Outcome	Cerebral atherosclerosis	FinnGen (R8)	342,499	282	342,217

GM taxa, gut microbiota taxa; NA, not available.

### 2.4 Instrumental Variable Selection

The IVs for the 211 GM taxa were selected using the following criteria: (1) Potential IVs were chosen based on SNPs associated with each taxon, with a statistical significance threshold of *p* < 1.0 × ${\mathrm{10}}^{-5}$ [16]. Only SNPs with F-statistics exceeding 10 were considered, while potential bias from weak IVs was disregarded if the F-statistic was greater than 10 [17]. (2) IVs were required to have independent and reduced linkage disequilibrium (LD), achieved by removing SNPs within a 10,000 kb range with a threshold of $r^{2}$< 0.001. SNPs with the lowest *p* values were preserved. (3) SNPs connected to the outcome with a *p* value greater than 1.0 × ${\mathrm{10}}^{-5}$ were excluded. (4) In cases of palindromic SNPs, harmonization was performed to exclude palindromic and incompatible SNPs. (5) To avoid potential pleiotropy, SNPs associated with confounding factors were identified using PhenoScanner V2 (http://www.phenoscanner.medschl.cam.ac.uk/). SNPs associated with atherosclerosis, such as obesity, hypertension, smoking, and other high-risk factors, were excluded [18, 19].

### 2.5 Statistical Analysis

In this study, various approaches, such as inverse variance-weighted (IVW), MR-Egger, weighted median, simple mode, and weighted mode were employed to investigate the potential causal link between GM and atherosclerosis in different vascular locations. To establish causality, the IVW technique (*p* < 0.05) was utilized as the primary technique since it combines Wald ratio estimates with a meta-analysis approach for each SNP, to provide a comprehensive assessment of the exposure impact on the outcome. The IVW approach is further enhanced by incorporating the Wald ratio estimator, which is a methodology grounded in meta-analysis. If there were no instances of horizontal pleiotropy, the results obtained from the IVW method would remain unbiased [20]. Once the demonstration of causality was accomplished through the IVW method, four additional MR techniques were employed to supplement the IVW findings: MR-Egger, weighted median, simple mode, and weighted mode [21, 22]. The foundation of the MR-Egger regression lies in the hypothesis that Instrument Strength Independent of Direct Effect (InSIDE) assesses the presence of pleiotropy by using the intercept term. If there is no evidence of horizontal pleiotropy and the intercept term equals zero, the results obtained from the MR-Egger regression align with those achieved through IVW [23]. Thus, to elucidate causality, the odds ratios (ORs) and 95% confidence intervals (CIs) were calculated and are presented. The significance level (*p* value) for the analyses was set at 0.05, with multiple testing corrections performed using the Bonferroni method. The significance threshold for each level was adjusted to divide 0.05 by n, where n represents the quantity of the different considered classifications.

### 2.6 Sensitivity Analysis

The level of conviction in the causal evidence is heightened when estimations demonstrate a persistent pattern across each MR method. To evaluate the steadfastness of the causal association, we undertook several sensitivity analyses. Initially, the MR-Egger intercept and MR-PRESSO global tests were employed to detect any potential horizontal pleiotropy [24, 25]. Additionally, a leave-one-out analysis was conducted to verify the reliability of the findings. Furthermore, the heterogeneity of the effect sizes resulting from the selected genetic IVs was quantified using the Cochran Q statistic.

R version 4.2.1 (R Foundation for Statistical Computing, Vienna, Austria) was used to complete all statistical analyses. MR analysis was performed using the TwosampleMR (version 0.5.6) packages in R [25, 26].

## 3. Results

### 3.1 Instrumental Variable

Upon filtration, 2686 SNPs were selected as IVs from a wide selection of 122,110 SNPs. These SNPs were categorized into five distinct classifications based on their phylum, class, order, family, and genus. Specifically, each phylum consisted of 116 IVs, each class comprised 215 IVs, each order encompassed 264 IVs, each family contained 462 IVs, and each genus included an impressive 1629 IVs.

### 3.2 MR Analysis

#### 3.2.1 Relationship between Gut Microbiota and Atherosclerosis

**Fig. 2.**
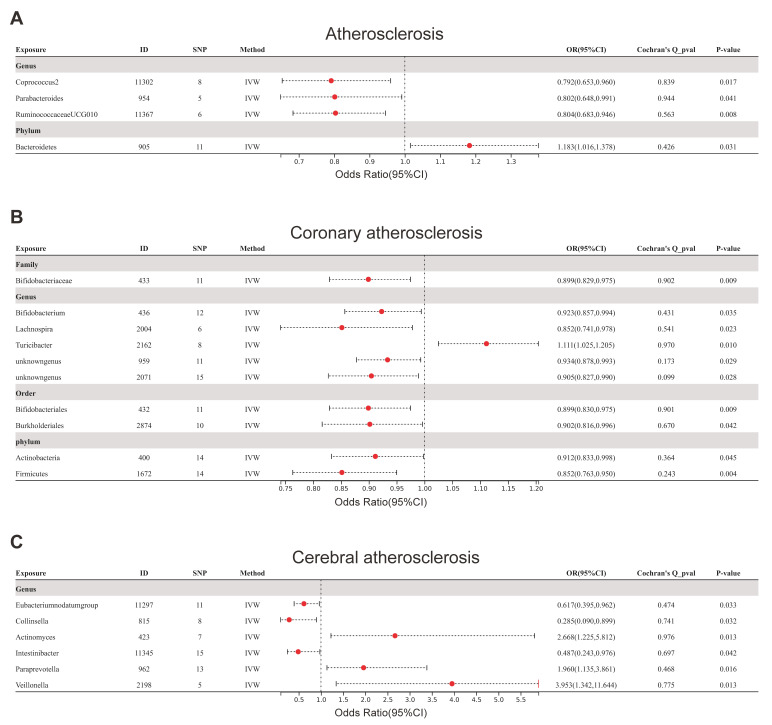
**Forest plots for all outcomes using the IVW method**. (A) Forest plot of the links between gut microbiota taxa and atherosclerosis. (B) Forest plot of the link between gut microbiota taxa and coronary atherosclerosis. (C) Forest plot of the link between gut microbiota taxa and cerebral atherosclerosis. SNP, single nucleotide polymorphism; IVW, inverse variance-weighted; OR, odds ratio; ID, identifier of gut microbiota.

In accordance with the evaluation conducted by the IVW, it was found that the phylum Bacteroidetes (ID: 905) were associated with an elevated susceptibility to atherosclerosis, whereas the genera Coprococcus2 (ID: 11302), Parabacteroides (ID: 954), and RuminococcaceaeUCG010 (ID: 11367) were associated with a decreased risk (Fig. 2A). However, it is noteworthy that the causal correlation between these gut microbiota taxa and atherosclerosis was rendered insignificant after the Bonferroni adjustment was applied. Furthermore, the results derived from Cochran’s Q test indicated an absence of heterogeneity.

#### 3.2.2 Relationship between Gut Microbiota and Coronary Atherosclerosis

The findings from further assessments conducted by IVW revealed that the genus Turicibacter (ID: 2162) was associated with an elevated susceptibility to coronary atherosclerosis, while the family Bifidobacteriaceae (ID: 433); genera: Bifidobacterium (ID: 436), Lachnospira (ID: 2004), unknown genus (ID: 959), unknown genus (ID: 2071); orders Bifidobacteriales (ID: 432), Burkholderiales (ID: 2874); phyla Actinobacteria (ID: 400), and Firmicutes (ID: 1672) were associated with a reduced risk of atherosclerosis (Fig. 2B). In addition, the results derived from Cochran’s Q test indicated an absence of heterogeneity. The phylum of Firmicutes (ID: 1672) (OR = 0.852 (0.763, 0.950), *p* = 0.004) remained linked to a lower risk of coronary atherosclerosis after Bonferroni adjustment.

#### 3.2.3 Relationship between Gut Microbiota and Cerebral Atherosclerosis

Ultimately, the outcomes indicated by the IVW technique demonstrated that the classifications Eubacterium nodatum (ID: 11297), Collinsella (ID: 815), and Intestinibacter (ID: 11345) were associated with a decreased likelihood of developing atherosclerosis. Conversely, the classifications Actinomyces (ID: 423), Paraprevotella (ID: 962), and Veillonella (ID: 2198) were associated with an increased risk of cerebral atherosclerosis (Fig. 2C). However, it is worth noting that the causality between these specific gut microbiota taxa and cerebral atherosclerosis lacked significance following Bonferroni adjustment. Additionally, the results derived from Cochran’s Q test indicated an absence of heterogeneity.

**Fig. 3.**
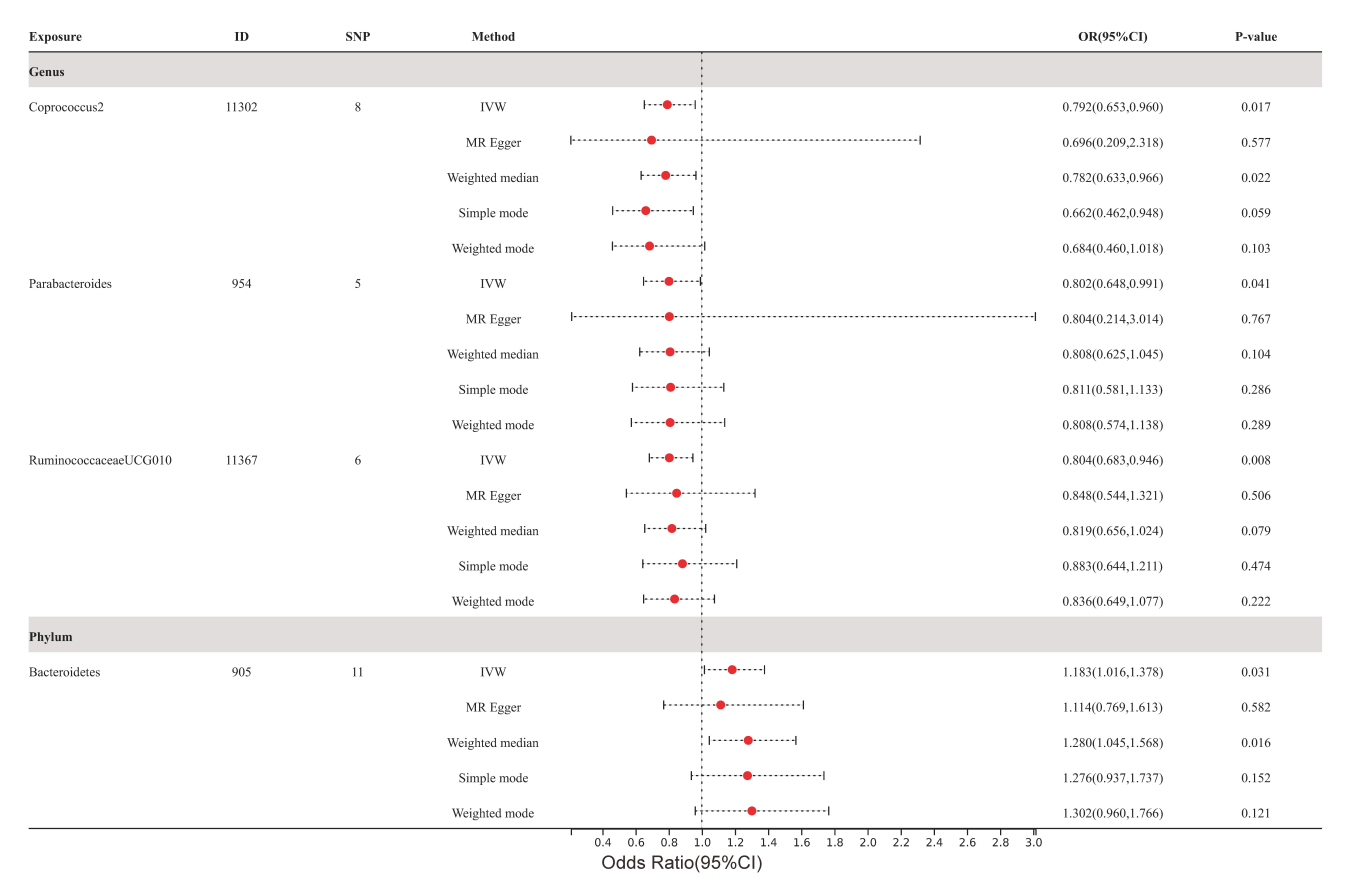
**Forest plots of atherosclerosis for all methods**. MR results for four gut microbiota taxa causality links to atherosclerosis. SNP, single nucleotide polymorphism; IVW, inverse variance-weighted; OR, odds ratio; MR, Mendelian randomization; ID, identifier of gut microbiota.

**Fig. 4.**
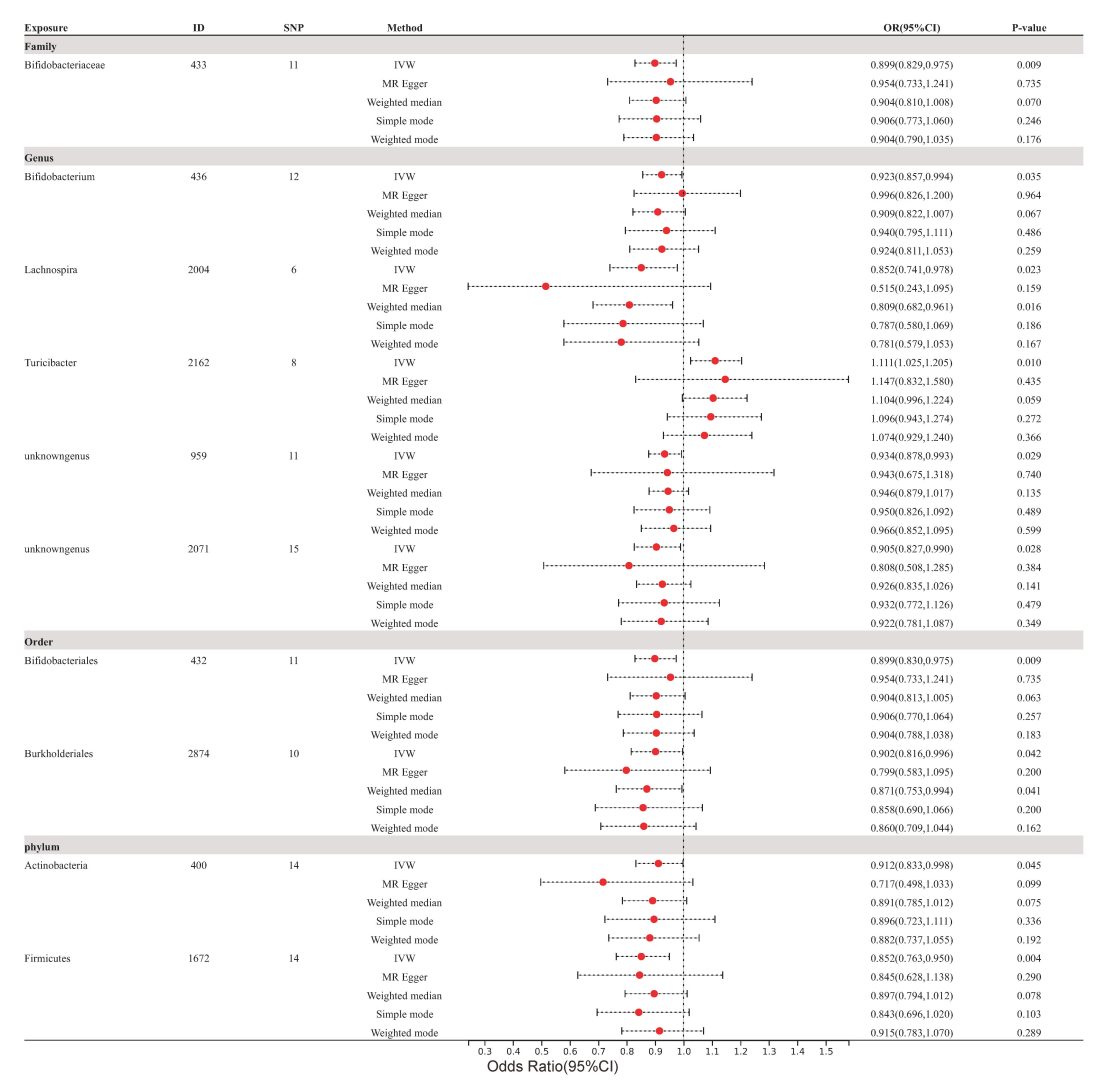
**Forest plots of coronary atherosclerosis for all methods**. MR results for 10 gut microbiota taxa causality links to coronary atherosclerosis. SNP, single nucleotide polymorphism; IVW, inverse variance-weighted; OR, odds ratio; MR, Mendelian randomization; ID, identifier of gut microbiota.

**Fig. 5.**
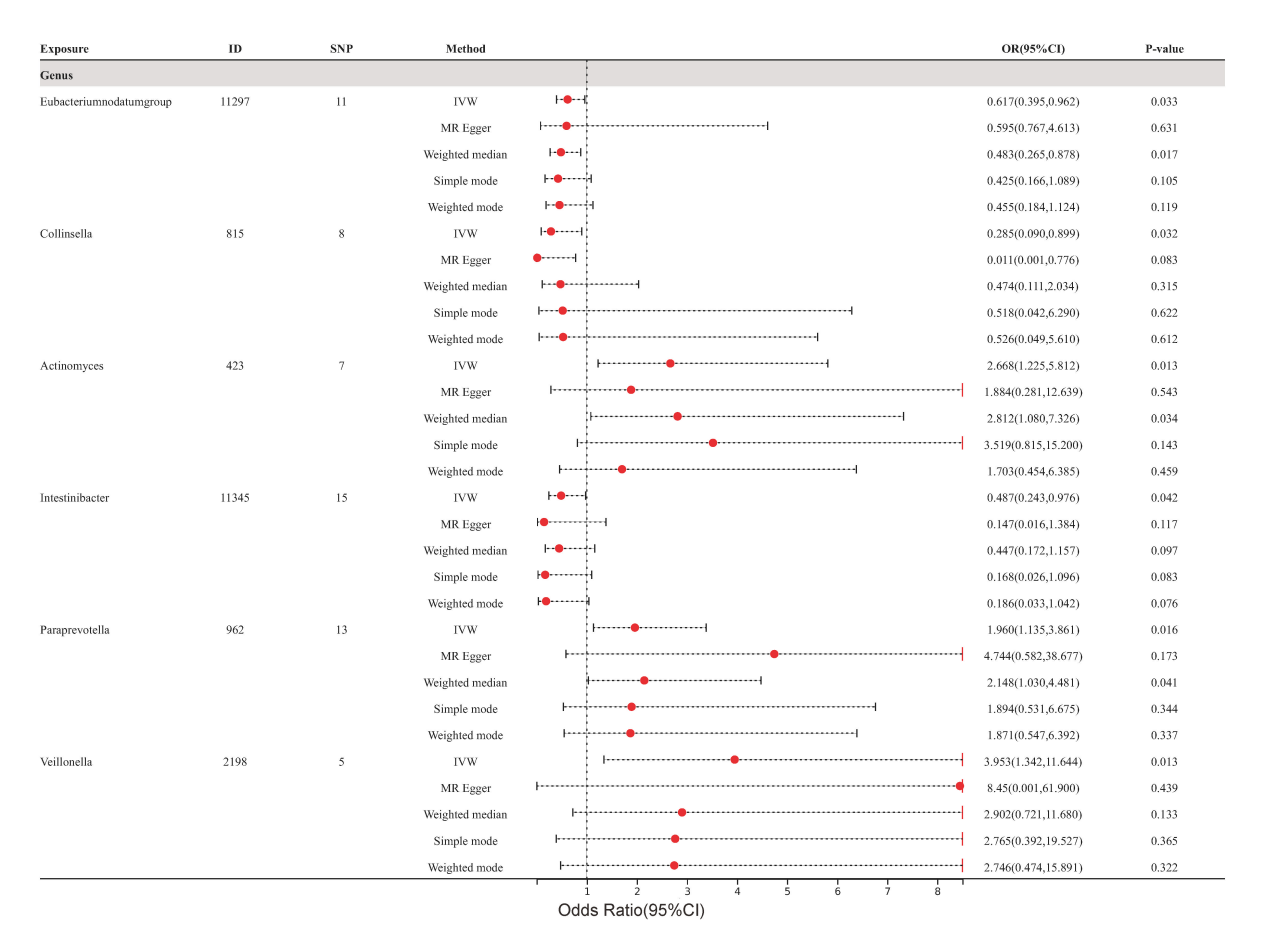
**Forest plots of cerebral atherosclerosis for all methods**. MR results for six gut microbiota taxa causality links to cerebral atherosclerosis. SNP, single nucleotide polymorphism; IVW, inverse variance-weighted; OR, odds ratio; MR, Mendelian randomization; ID, identifier of gut microbiota.

The impact of these specific gut microbiota taxa on the development of atherosclerosis (as depicted in Fig. 3), coronary atherosclerosis (as depicted in Fig. 4), and cerebral atherosclerosis (as depicted in Fig. 5) was assessed through the implementation of four supplementary methodologies: MR-Egger, weighted median, simple mode, and weighted mode. The findings were consistent with those obtained by IVW analysis (refer to **Supplementary Figs. 1,2,3**).

### 3.3 Sensitivity Analysis

The absence of horizontal pleiotropy was indicated by outcomes of *p* > 0.05, which were obtained from both the MR-Egger intercept test and the MR-PRESSO global test (Tables 2,3,4). Through the leave-one-out analysis, it was demonstrated that there were no significant alterations in the risk estimates for the genetically estimated risks, thereby suggesting that the overall outcome remained robust, even when removing any single SNP (**Supplementary Figs. 4,5,6**).

**Table 2.** **Sensitivity analysis for the correlation between atherosclerosis and gut microbiota**.

Gut microbiota taxa	MR-Egger intercept test			MR-PRESSO global test	
	Egger value	Standard error	*p* value	RSS obs	*p* value
Coprococcus2 (ID: 11302)	0.010	0.048	0.839	16.086	0.126
Parabacteroides (ID: 954)	–0.001	0.058	0.997	1.089	0.950
Ruminococcaceae (ID: 11367)	–0.004	0.016	0.813	5.175	0.636
Bacteroidetes (ID: 905)	0.004	0.013	0.731	12.831	0.424

MR, Mendelian randomization; ID, identifier of gut microbiota; RSS obs, observed residual sum of squares.

**Table 3.** **Sensitivity analysis for the correlation between coronary atherosclerosis and gut microbiota**.

Gut microbiota taxa	MR-Egger intercept test			MR-PRESSO global test	
	Egger value	Standard error	*p* value	RSS obs	*p* value
Bifidobacteriaceae (ID: 433)	–0.004	0.010	0.653	5.495	0.938
Bifidobacterium (ID: 436)	–0.007	0.008	0.406	13.113	0.461
Lachnospira (ID: 2004)	0.031	0.023	0.255	6.112	0.555
Turicibacter (ID: 2162)	–0.003	0.017	0.849	2.461	0.985
Unknown genus (ID: 959)	–0.001	0.019	0.952	17.199	0.224
Unknown genus (ID: 2071)	0.009	0.187	0.635	24.097	0.124
Bifidobacteriales (ID: 432)	–0.005	0.009	0.653	5.495	0.920
Burkholderiales (ID: 2874)	0.009	0.011	0.449	8.161	0.707
Actinobacteria (ID: 400)	0.015	0.011	0.208	16.377	0.352
Firmicutes (ID: 1672)	0.001	0.011	0.961	26.785	0.066

MR, Mendelian randomization; ID, identifier of gut microbiota; RSS obs, observed residual sum of squares.

**Table 4.** **Sensitivity analysis for the correlation between cerebral atherosclerosis and gut microbiota**.

Gut microbiota taxa	MR-Egger intercept test			MR-PRESSO global test	
	Egger value	Standard error	*p* value	RSS obs	*p* value
Eubacterium nodatum (ID: 11927)	0.005	0.152	0.973	11.488	0.504
Actinomyces (ID: 423)	0.043	0.109	0.711	1.973	0.967
Collinsella (ID: 815)	0.251	0.161	0.170	5.624	0.748
Intestinibacte (ID: 11345)	0.101	0.091	0.291	12.504	0.716
Paraprevotella (ID: 962)	–0.097	0.019	0.411	13.575	0.504
Veillonella (ID: 2198)	–0.727	0.929	0.491	2.851	0.784

MR, Mendelian randomization; ID, identifier of gut microbiota; RSS obs, observed residual sum of squares.

## 4. Discussion

The MR method was utilized to investigate a potential causal relationship between various GM taxa and atherosclerosis across three distinct vascular sites. The findings revealed a total of 20 causal connections, with one demonstrating strong causality and the remaining 19 showing nominal causality.

This study also unveiled a strong causal relationship following Bonferroni correction, which indicated that the Firmicutes phylum (ID: 1672) (OR = 0.852 (0.763, 0.950), *p* = 0.004) markedly mitigates the risk of coronary atherosclerosis. Therefore, this MR analysis reveals the protective function of the Firmicutes phylum in relation to coronary atherosclerosis. Among the assemblages prevalent in the human gut microbiota, the Firmicutes phylum stands out as a dominant faction [27]. The abundance of Eubacterium and Roseburia, which belong to the Firmicutes phylum, in the gut microbiota of patients with atherosclerosis was found to be reduced compared to the healthy control group [8, 28, 29]. Our findings align with those from other early studies and corroborate the assertion that patients with coronary atherosclerosis have a low abundance of the phylum Firmicutes in their intestinal microbiota. Growing evidence has demonstrated the impact that gut microbiome can have on cardiovascular health [30]. The gut microbiota may exert its influence on atherosclerosis through various mechanisms, with gut metabolites being regarded as one of the primary mechanisms impacting atherosclerosis [31]. Although research on the phylum Firmicutes is relatively limited, some observational studies and animal models have reported on the impact that metabolites produced by gut microbiota belonging to the phylum Firmicutes have on the occurrence and progression of atherosclerosis. For example, bacteria in the phylum Firmicutes, Clostridium, and Peptostreptococcaceae, are capable of producing IPA, a microbial metabolite derived from tryptophan. IPA has been found to downregulate the expression of miR-142-5p in macrophages, which promotes the transportation of extracellular cholesterol. Low levels of IPA have been associated with risk factors of coronary atherosclerosis, and this study also uncovered the potential of using IPA supplementation to suppress the progression of arterial atherosclerosis [32]. Meanwhile, Karlsson *et al*. [28] discovered that bacteria belonging to the phylum Firmicutes, specifically Ruminococcaceae spp, Eubacterium, and Roseburia, are capable of producing short-chain fatty acids (SCFAs). It has been observed that the abundance of these bacterial populations is reduced in patients with atherosclerosis [8, 28, 29], and SCFAs have been found to possess anti-inflammatory properties toward certain epithelial cells. This may potentially slow down the progression of atherosclerosis [33]. Furthermore, Rath *et al*. [34] found that Clostridia bacteria degrade nutrients such as phosphatidylcholine to produce TMAO, while the levels of TMAO are associated with the occurrence of atherosclerosis. However, current research has yet to confirm a causal relationship between them [31].

It is noteworthy that the criteria for Bonferroni correction are excessively stringent, sacrificing some statistical efficiency and potentially resulting in false negative outcomes. Initial associations between 19 gut microbiota taxa were observed in our study; however, these associations disappeared after applying the Bonferroni correction. This may be attributed to the possibility that a single microbiota genus might not play as substantial a role in disease as previously postulated, in relation to the gut microbiome and cardiovascular disease, which are influenced by multiple factors [35]. Furthermore, our results indicated several gut microbiota taxa with preliminary causal links, thereby corroborating prior research discoveries. For example, Karlsson *et al*. [28] observed decreased levels of Eubacterium in the gut microbiota of atherosclerosis patients compared to healthy controls. Likewise, Liu *et al*. [29] found that Ruminococcaceae were reduced in atherosclerosis patients compared to healthy controls. Although these gut microbiota taxa exhibit only preliminary causal associations at the genus level, further investigations into the collaboration and interaction among diverse gut microbiota taxa remain worthwhile.

Moreover, it is imperative to acknowledge the inherent constraints in our investigation. Primarily, we employed a rather lenient criterion (*p* < 1 × ${\mathrm{10}}^{-5}$) to filter the IVs owing to the minuscule count of IVs that satisfied the stringent threshold (*p* < 5 × ${\mathrm{10}}^{-8}$). Furthermore, to augment the study’s validity, future analyses necessitate the utilization of a larger sample size of GWAS data due to the relatively sparse frequency of cerebral atherosclerosis cases. Lastly, bearing in mind the predominantly European heritage of our participants, it is essential to exercise caution when generalizing these findings to other populations.

Overall, these MR research findings align with the majority of the previously published studies. These studies collectively suggest that the impact on atherosclerosis by the phylum Firmicutes is primarily mediated through its metabolites. Thus, increasing the abundance of Firmicutes in the gut microbiota may serve as a protective measure against atherosclerosis. However, further experimental validation is required to confirm this hypothesis. The results of our MR study can provide a confident direction for future research endeavors.

## 5. Conclusions

Conclusively, a total of 20 nominal connections and a strong causal relationship were successfully identified by conducting a meticulous MR analysis on the risk of distinct GM taxa in relation to atherosclerosis across three diverse vascular sites. Notably, the phylum Firmicutes has been scientifically linked to a reduced occurrence of coronary atherosclerosis, as the gathered data reveals. To shed light on the significant beneficial impact of Firmicutes on coronary atherosclerosis, along with its distinct protective mechanisms, conducting future RCT studies is imperative. However, these findings present a fresh perspective on potential strategies for the prevention and treatment of atherosclerosis by targeting gut microbiota and exploring prospective therapeutic targets for this condition.

## Data Availability

Publicly available datasets were analyzed in this study. This data can be found at: https://mibiogen.gcc.rug.nl/, https://www.finngen.fi/fi.

## Supplementary Material

Supplementary material associated with this article can be found, in the online version, at https://doi.org/10.31083/j.rcm2502041.
