# Supplementary material for: Gut Microbiome and Atherosclerosis: A Mendelian Randomization Study
Source: Rev Cardiovasc Med. 2024 Jan 29;25(2):41. doi: 10.31083/j.rcm2502041 (PMC11263158; doi:10.31083/j.rcm2502041)
Supplement: Supplementary file 1 [file 2153-8174-25-2-041-s1.zip › 2153-8174-25-2-041-s1/Supplementary Fig. 3.pdf]

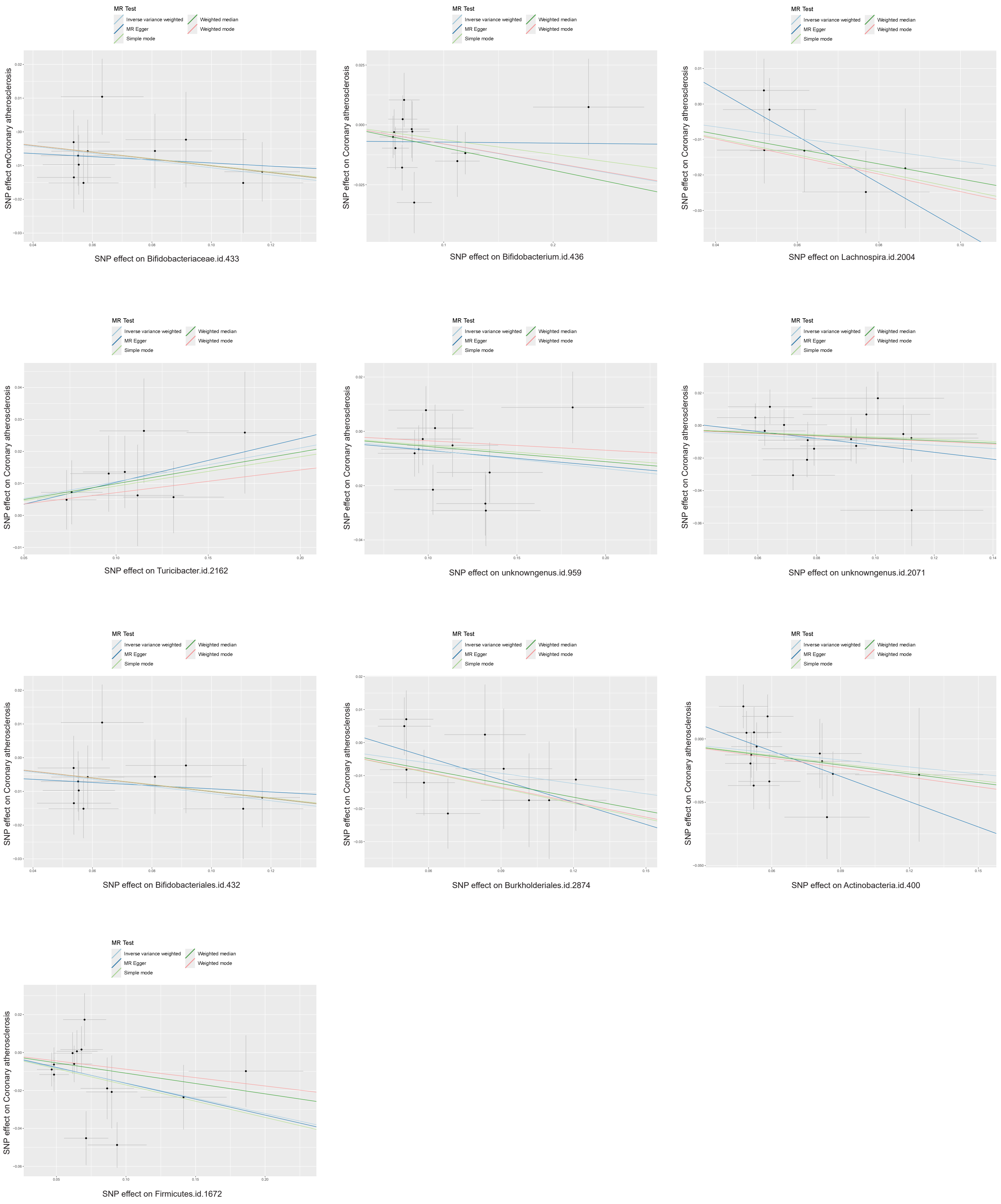

Supplementary Fig. 3. Scatter plot of MR results. scatter plot of genetic correlations of 10 GM taxa and Coronary atherosclerosis using different MR methods.
