# Supplementary material for: Gut Microbiome and Atherosclerosis: A Mendelian Randomization Study
Source: Rev Cardiovasc Med. 2024 Jan 29;25(2):41. doi: 10.31083/j.rcm2502041 (PMC11263158; doi:10.31083/j.rcm2502041)
Supplement: Supplementary file 1 [file 2153-8174-25-2-041-s1.zip › 2153-8174-25-2-041-s1/Supplementary Fig. 4.pdf]

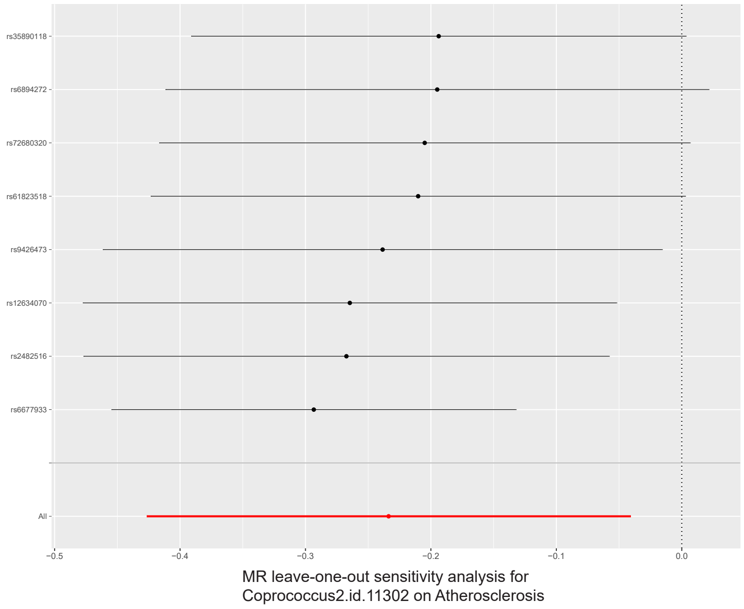

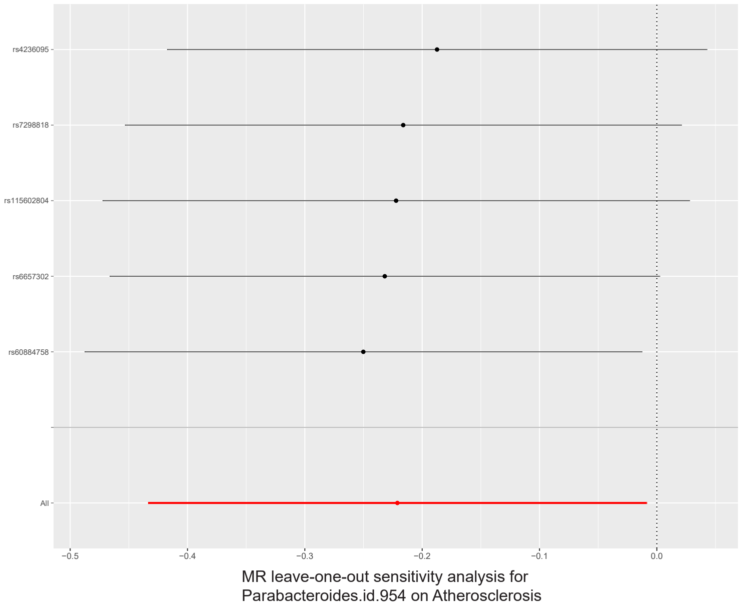

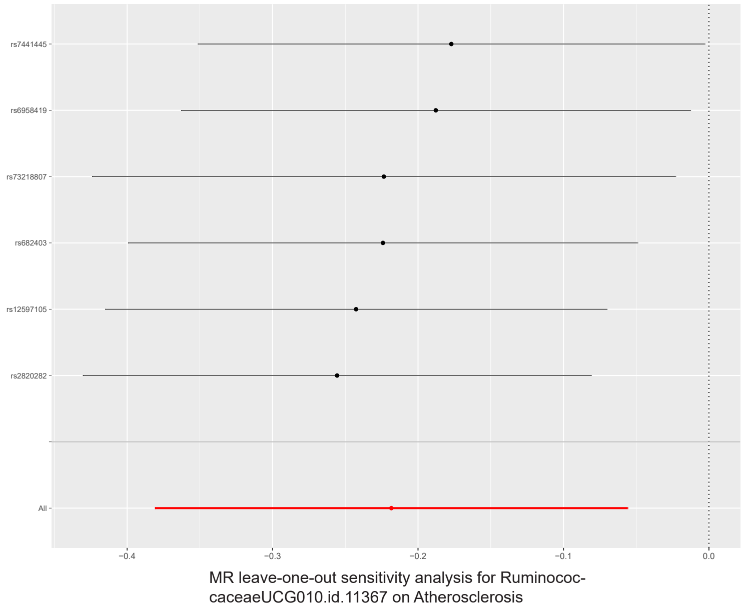

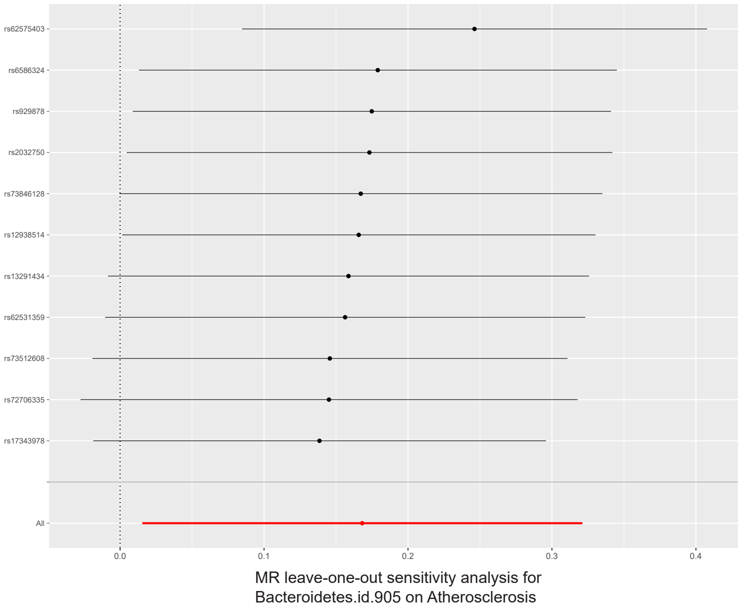

Supplementary Fig. 4. Leave-one-out analysis for 4GM taxa on Atherosclerosis(excluding cerebral, coronary and PAD).
