# Supplementary material for: Gut Microbiome and Atherosclerosis: A Mendelian Randomization Study
Source: Rev Cardiovasc Med. 2024 Jan 29;25(2):41. doi: 10.31083/j.rcm2502041 (PMC11263158; doi:10.31083/j.rcm2502041)
Supplement: Supplementary file 1 [file 2153-8174-25-2-041-s1.zip › 2153-8174-25-2-041-s1/Supplementary Fig. 6.pdf]

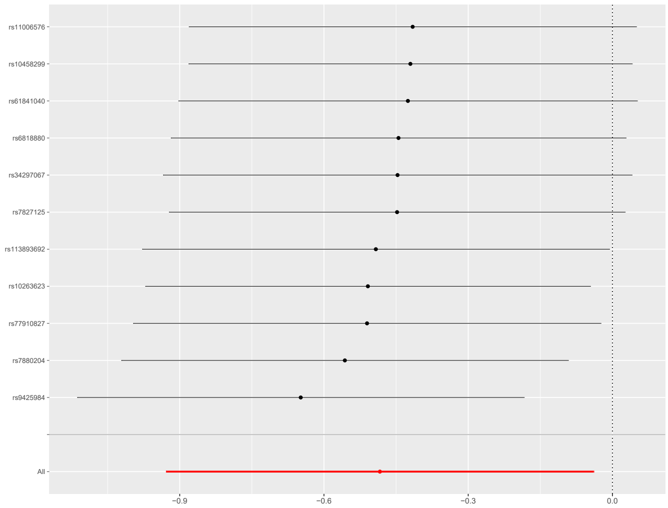

MR leave-one-out sensitivity analysis for Eubacteriumno-datumgroup.id.11297 on Cerebral Atherosclerosis

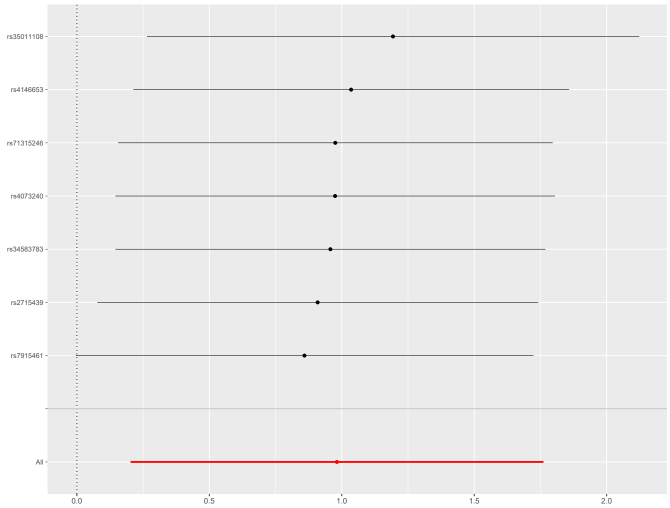

MR leave-one-out sensitivity analysis for Eubacterium-noActinomyces.id.423 on Cerebral Atherosclerosis

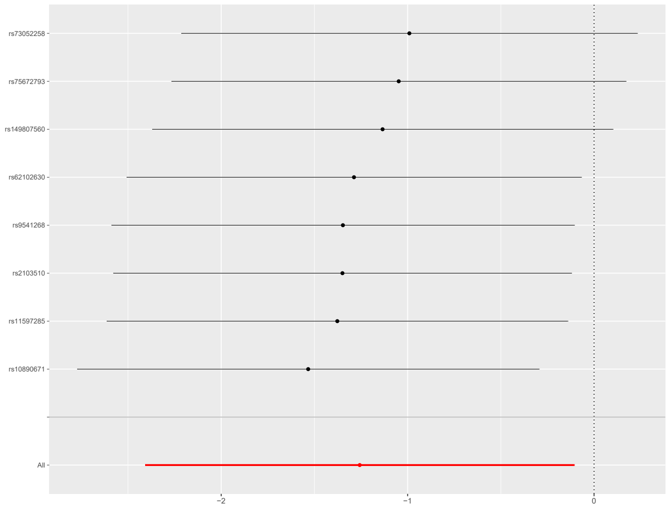

MR leave-one-out sensitivity analysis for Collinsella.id.815 on Cerebral Atherosclerosis

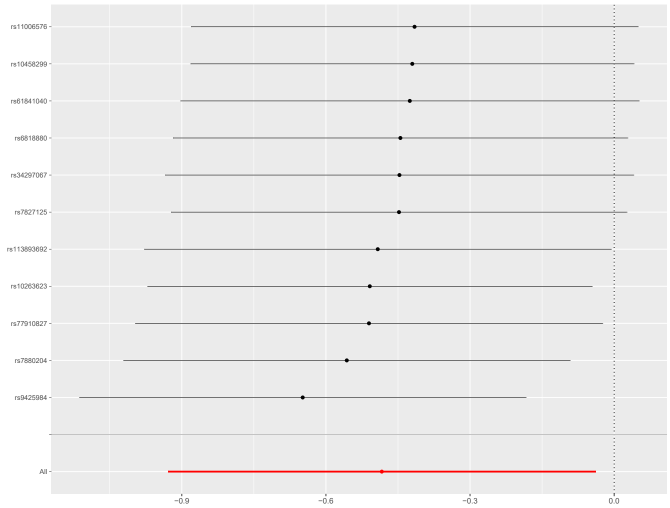

MR leave-one-out sensitivity analysis for Intestinibacter.id.11345 on Cerebral Atherosclerosis

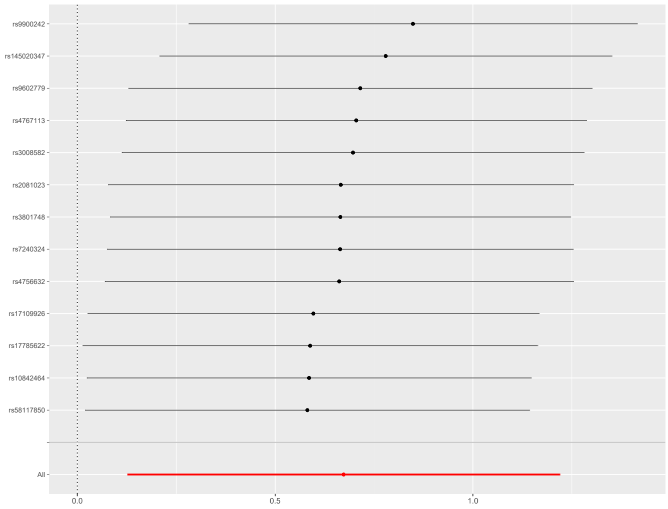

MR leave-one-out sensitivity analysis for Paraprevotella.id.962 on Cerebral Atherosclerosis

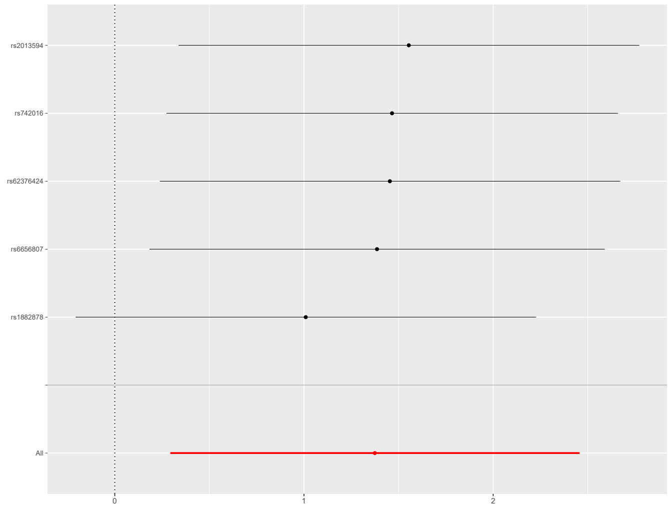

MR leave-one-out sensitivity analysis for Veillonella.id.2198 on Cerebral Atherosclerosis

Supplementary Fig. 6. Leave-one-out analysis for 6GM taxa on Cerebral Atherosclerosis.
