# Supplementary figures and images for: Gut Microbiome and Atherosclerosis: A Mendelian Randomization Study
Source: Rev Cardiovasc Med. 2024 Jan 29;25(2):41. doi: 10.31083/j.rcm2502041 (PMC11263158; doi:10.31083/j.rcm2502041)

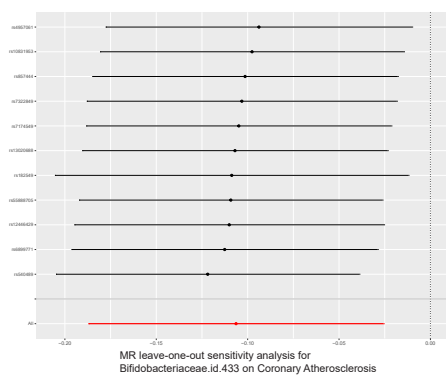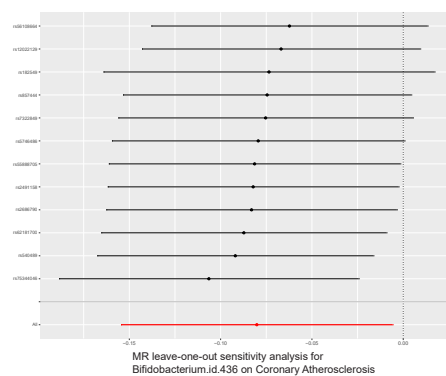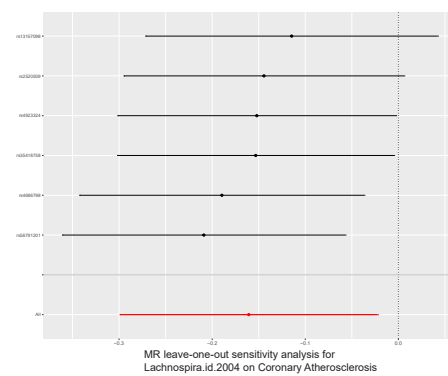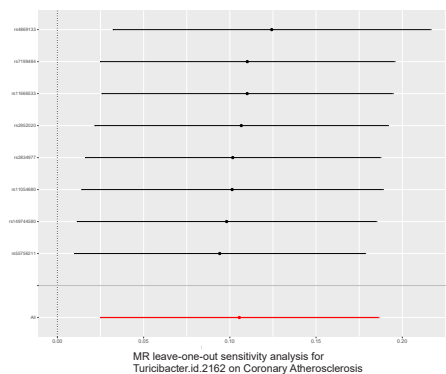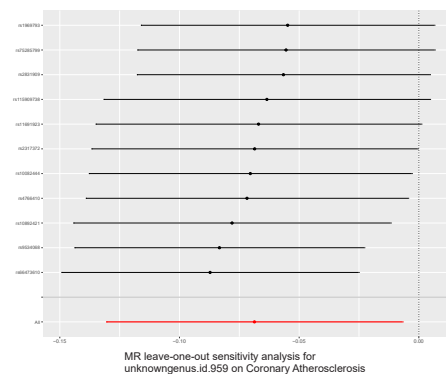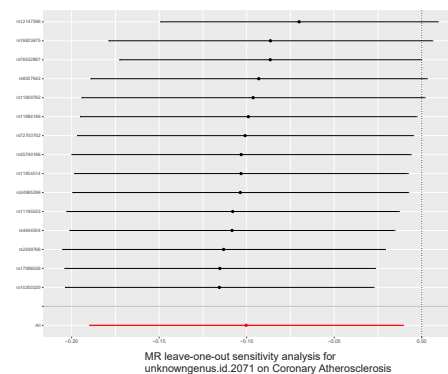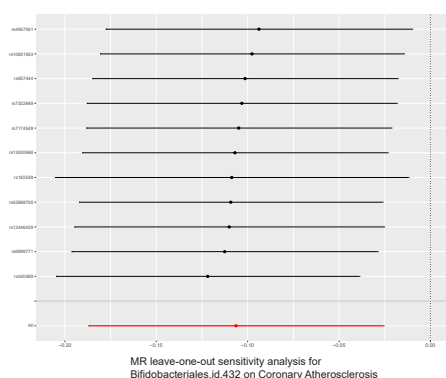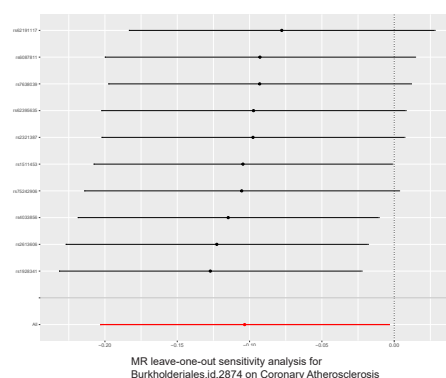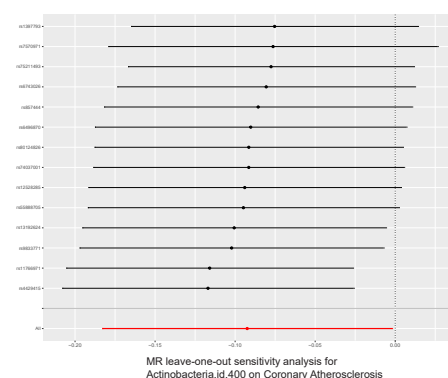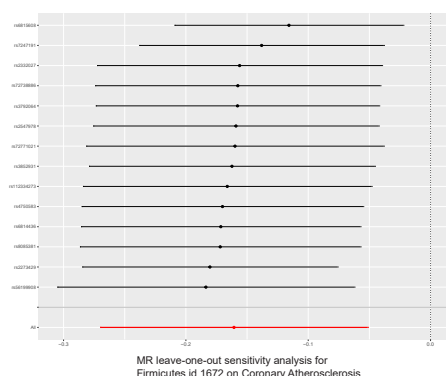

**Supplementary Fig. 5.** Leave-one-out analysis for 10GM taxa on Coronary Atherosclerosis.

Supplement: Supplementary file 1 [file 2153-8174-25-2-041-s1.zip › 2153-8174-25-2-041-s1/Supplementary Fig. 5.pdf]
